# Walleye Dermal Sarcoma Virus: Molecular Biology and Oncogenesis

**DOI:** 10.3390/v2091984

**Published:** 2010-09-22

**Authors:** Joel Rovnak, Sandra L. Quackenbush

**Affiliations:** Department of Microbiology, Immunology and Pathology, Colorado State University, Fort Collins, 80523, USA; E-Mail: joel.rovnak@colostate.edu (J.R.)

**Keywords:** retrovirus, epsilonretrovirus, oncogenesis, walleye dermal sarcoma virus, fish, retroviral cyclin, RACK1, apoptosis, mitochondria

## Abstract

Retroviruses have been detected in most vertebrate species and are etiologic agents of a variety of neoplastic diseases. The study of retroviruses has been instrumental in uncovering the molecular mechanisms responsible for oncogenesis. Retroviruses have been isolated from three neoplastic diseases in fish, two of which affect the dermis and regress naturally coincident with spawning. This feature provides a unique model to study mechanisms of tumor development and regression. Three complex retroviruses, isolated from walleye (*Sander vitreus*) with dermal sarcoma and epidermal hyperplasia, are the members of the newest retroviral genus, *Epsilonretrovirus*. Three accessory proteins, encoded by walleye dermal sarcoma virus (WDSV), function in the regulation of host and viral gene expression and cell cycle, alter cell-signaling pathways to promote cell proliferation and block apoptosis, and, finally, induce apoptosis through dissipation of the mitochondrial membrane potential.

## 1. Introduction

The identification of retroviruses as etiologic agents of a variety of neoplasms began in the early 20th century. The study of these viruses has been instrumental in uncovering molecular mechanisms of tumor development. Exogenous retroviruses have been isolated from many vertebrates and are classified into two subfamilies, *Orthoretrovirinae* and *Spumaretrovirinae.* The *Orthoretrovirinae* are further broken down to six genera, *Alpharetrovirus, Betaretrovirus, Gammaretrovirus, Deltaretrovirus, Epsilonretrovirus,* and *Lentivirus*, based largely on highly conserved regions within the reverse transcriptase or “*pol*” gene. The most recent genus added to the *Retroviridae* is the *Epsilonretroviruses*, which includes three fish retroviruses: walleye dermal sarcoma virus (WDSV) and walleye epidermal hyperplasia viruses type 1 and type 2 (WEHV-1, WEHV-2 (www.ICTVonline.org). Two additional viruses, perch epidermal hyperplasia virus types 1 and 2 (PEHV-1, PEHV-2), are likely members, but their sequence is incomplete [1]. The exogenous piscine retroviruses, snakehead retrovirus (SnRV) and salmon swimbladder sarcoma-associated virus (SSSV), have not yet been assigned to a specific genus.

Exposure to chemical carcinogens and infection by herpesviruses has also been implicated in proliferative diseases of fish. The specific association of retroviral infection with proliferative lesions in fish is based on the presence of retrovirus-like particles and reverse transcriptase activity in neoplastic tissue [2]. To date, the complete sequence of five exogenous retroviruses, four of which are associated with neoplastic diseases in fish, have been determined [3–7]. SSSV was first identified in Atlantic salmon (*Salmo salar*) in 1976 from a commercial fish farm and is associated with neoplasia of the swimbladder [8,9]. The SnRV was isolated from cell culture and any association with disease is currently unknown [10]. This review will focus on walleye retroviruses and their associated neoplastic diseases with an emphasis on walleye dermal sarcoma virus.

## 2. Epsilonretroviruses and associated diseases

### 2.1. Disease, pathology and transmission

Retroviruses from two proliferative skin lesions in walleye (*Sander vitreus*), walleye dermal sarcoma (WDS) and walleye epidermal hyperplasia (WEV), have been isolated and their sequence determined [3,5,6,11–13]. These proliferative diseases were first reported by Walker in 1969 in walleye collected from Oneida Lake in New York State and have been reported to occur elsewhere in North America [14–16]. Upwards of 27% of walleye are affected with WDS and 10% with WEH annually in Oneida Lake [14,16–18]. The most interesting and defining feature of these proliferative diseases is their seasonal cycle [19]. The highest incidence of disease occurs throughout the late fall until the spring spawning period at which time the lesions naturally regress. During the summer months, lesions are very rarely observed [20].

WEH lesions are broad, flat, translucent plaques of thickened epidermis that range in size from 2 to 3 mm up to 50 mm in diameter. Histologically, WEH consists of localized hyperplasia of Malpighian cells with frequent mitotic figures [14–16].

**Figure 1.**
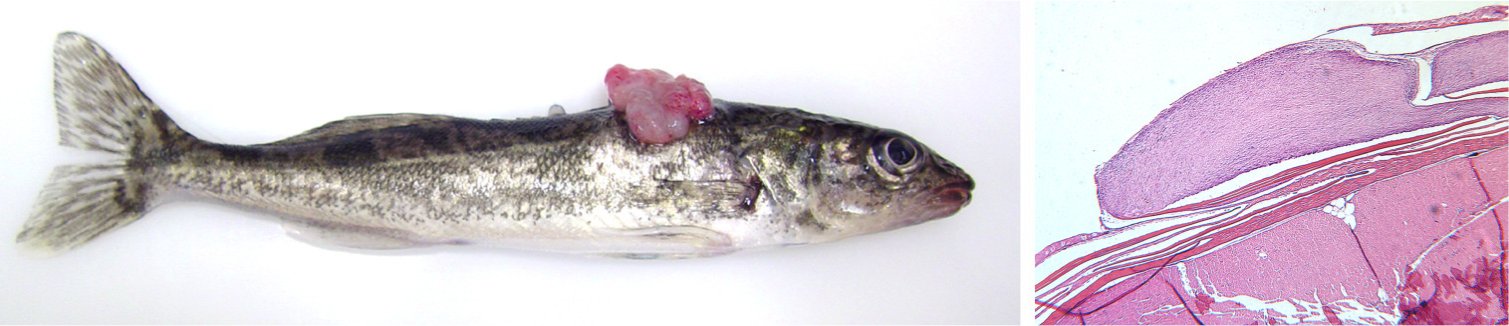
Experimental transmission of walleye dermal sarcoma with cell-free filtrate from regressing tumors. Left: Gross pathology of tumors. An individual tumor and a large mass of coalescing tumors are visible. Right: Histopathology of walleye dermal sarcoma.

WDS are cutaneous mesenchymal neoplasms that are randomly distributed on the fish, arise from the superficial surface of the scales and range in size from 0.2–1.0 cm in diameter (Figure 1) [14–16,21]. Multiple tumors often coalesce. Histologically, the tumors are non-encapsulated nodular masses consisting of interwoven bundles and whorls of fibroblast cells that abut the epidermis. Mitotic figures are rare and the overlaying epidermis is often ulcerated in the regressing tumors [21]. Perivascular accumulation of lymphocytes and aggregates of inflammatory cells within the dermis surrounding the mass have been observed in some tumors [2,21,22]. Tumors have not been observed in internal organs [21,23]. Rare cases of invasive WDS have been documented in adult wild fish where the tumor tissue invaded below the dermis into the muscle and bone of the operculum [24].

The observation of type C retroviral particles in tumors by electron microscopy provided the first evidence for retroviral infection associated with proliferative lesions in walleye [14,18]. To demonstrate a viral etiology for walleye dermal sarcoma, 12-week old walleye fingerlings were inoculated with cell-free material prepared from regressing tumors collected during the spring spawning run [25]. Transmission of disease is very efficient: Within 14 weeks post infection, 87% of experimentally infected fish developed tumors that were grossly and histologically identical to those observed on naturally affected adult walleyes (Figure 1) [25]. Experimental transmission of disease is successful with intramuscular injection, oral gavage or topical application of infectious material [26]. A fundamental finding from transmission studies was the inability of homogenates of developing tumors to transmit disease. These tumors were collected from fish in the late fall, and the lack of transmission is due to the absence of infectious virus [27].

Locally invasive tumors were observed in experimental transmission studies and their incidence was correlated with the age of fish at the time of infection [26,28]. All fish infected at 6–8 weeks of age developed invasive tumors whereas only 2% of fish infected at 12 weeks of age developed invasive tumors [26,28]. No fish infected at 52 weeks of age developed invasive tumors. Poor transmission of disease was observed with cell-free material from naturally occurring invasive tumors, which likely reflects the low levels of infectious virus present in these tumors [24]. Dermal sarcoma is also experimentally transmissible to sauger (*Stizostedion canadense)* and yellow perch (*Perca flavescens*) [29,30]. Transmission of WEH to walleye fingerlings has also been achieved with cell-free material from lesions collected in the spring [17].

Based on the observation that tumor development and regression is seasonal in nature, the experimental transmission model of WDSV was used to address any correlation between water temperature and tumor regression. Fish were infected and held at 15 °C. Five months after the development of tumors, fish were randomly assigned to temperature treatment groups of 10 °C, 15 °C or 20 °C for an additional 5 months. Tumor regression occurred in 3% of fish held at 10 °C, 28% of fish held at 15 °C and 32% of fish held at 20 °C, suggesting that increases in water temperature may affect regression [31].

Transmission of WDSV in nature is believed to occur through contact with water containing infectious virus or by direct contact of walleye during the spring spawning run, a time when virus expression in tumors is plentiful and fish are congregating in high numbers [32]. Studies conducted at the Oneida Lake Fish Cultural Station from 1995 through 2003 on the prevalence of WDS in different age classes of walleye indicated that fish only develop tumors for one season and remain tumor-free in following years, implicating a role for an immune response in tumor regression [33]. The experimental transmission and regression model system was used to address this possibility. Fish previously infected with virus were rechallenged and monitored for the appearance of new tumors. The incidence of new tumors on these fish was significantly lower than that in naïve fish infected with the same inoculum [34]. These studies also indicated that most walleye will develop WDS in their lifetime after the age of sexual maturity and first participation in the spring spawning.

### 2.2. Molecular characterization of Epsilonretroviruses

WDS and WEH transmission was demonstrated with cell free tumor homogenates and type C retroviral particles have been observed in tumors [14,17,25]. Further characterization of these viruses was hindered by the lack of a cell culture system for virus propagation. Therefore, purified virus from tumors was utilized in the initial molecular characterization studies [12,13]. Virus particles were purified from tumors by sucrose density gradient centrifugation [12,13]. Reverse transcriptase activity was detected in pooled fractions with a density of 1.18 gm/ml and electron microscopy was used to visualize viral particles. RNA was prepared from the sucrose banded virions and used for the synthesis of a cDNA hybridization probe [13].

**Figure 2.**
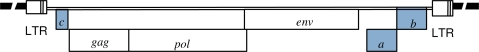
Organization of the WDSV genome.

WDSV was originally cloned from regressing tumor DNA in 1990 and found to be 12.7 kb in length [12,13]. Sequence analysis identified, in addition to *gag*, *pro*, *pol* and *env*, three open reading frames, designated *orf a*, *orf b* and *orf c*, that encode viral accessory proteins (Figure 2) [5]. A productively infected cell line, STEC, was established from a regressing tumor in 2003, approximately 13 years after the harvest of the tumor used for the original WDSV sequence [3]. The sequence of WDSV present in the STEC cell line revealed a total of 27 point mutations over the entire genome (12,706 bp) compared to the previous sequence. Only six of these mutations altered amino acid sequences: one in the amino terminus of RT, four in the surface domain (SU) of envelope, and one in the Orf B protein [3]. There were no variations in the LTR or upstream untranslated region of the genome. A significant sequence variation was identified in the transmembrane region (TM) of *env*: the insertion of one guanine and one cytosine residue at nt 9340 and nt 9347, respectively. These insertions result in a frameshift and the generation of a stop codon at nt 9451 compared with the stop at nt 9649 in the original WDSV molecular clone. The result is an alteration of the 37 carboxy-terminal amino acids and shortening of the envelope protein TM by 63 amino acids (corrected *env* sequence GenBank accession no. EF428979) [3,5]. Resequencing of the original WDSV clone identified these same insertions indicating that they are not variations from the 1990 isolate. The revised envelope protein has at least two predicted membrane-spanning regions [3,35,36]. These transmembrane helices lie between amino acids (a.a.) 850 and 874 and a.a. 1045 and 1069 with an intervening cytoplasmic loop. Budding virus was observed within STECs adjacent to internal membranes or cisternae, including mitochondrial membranes, and approximately one half of the infectious virus produced by these cells remains within the cell. Examination of the envelope sequence identified a consensus arginine-based endoplasmic reticulum (ER) localization signal, IRRER (a.a. 880–884), in the predicted cytoplasmic loop adjacent to the helix terminating at a.a. 874. This signal may serve to retain the envelope protein at internal membranes and cause the virus to bud internally in a manner similar to that of the spumaretroviruses, which use a KKXX motif for ER retention. Such a mechanism may be critical to the stability of infectious virus in an aquatic environment.

The results of WDSV sequence analysis made possible investigations of virus expression in tumors. Of particular interest is the quantitative and qualitative differences noted in the extent of virus transcripts and viral DNA in developing and regressing tumors. Developing tumors contain approximately one copy of viral DNA per cell, whereas cells of regressing tumors have 10–50 copies, most of which are unintegrated [3,5,12,27,37]. During the period of tumor growth, only the *orf a* and *orf b* subgenomic transcripts are expressed, whereas the spliced *env* transcript and full-length genomic RNA along with a variety of subgenomic spliced transcripts are expressed only in regressing tumors [12,27,37,38]. These results explain the inability to transmit disease with homogenates of developing tumors, due to the lack of expression of the viral genome, and implicate the products of *orf a* and *orf b* in tumor cell proliferation [27,38].

The two retroviruses that are associated with walleye epidermal hyperplasia, WEHV-type 1 and WEHV-type 2 were cloned by RT-PCR using degenerate *pol* primers followed by 5′ and 3′ RACE and the generation of a lambda genomic library [39,40]. Sequence analysis determined that WEHV-1 and WEHV-2 are 13.0 and 13.1 kb in length and have the same genomic organization as WDSV [5,6]. All the walleye retroviruses utilize histidyl-tRNA as a primer for initiation of minus-strand synthesis. One to three copies per cell of integrated proviral DNA is found in epidermal hyperplasia lesions [11]. WEHV also exhibits temporal gene expression in lesions collected in the fall and spring, similar to that of WDSV. The function of the WEHV accessory gene proteins has not been extensively studied.

Hyperplasia lesions similar to that described in walleye are also found on yellow perch in Oneida Lake and these lesions are associated with two new retroviruses tentatively identified as perch epidermal hyperplasia virus types 1 and 2 [1,14]. Based on partial sequence, the perch hyperplasia viruses are distinct from the walleye viruses, but their genome organization is likely similar to them.

### 2.3. WDSV accessory protein function

**Figure 3.**
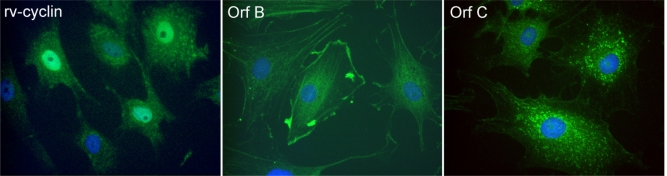
Expression of WDSV accessory proteins in cells derived from a naturally occurring spring tumor. Cells were labeled with rabbit antisera reactive to the indicated WDSV proteins and FITC-conjugated goat anti-rabbit IgG (green). Nuclei were stained with DAPI (blue). Magnification x400.

Immunofluorescence was used to visualize the expression and localization of the predicted WDSV accessory proteins in naturally infected tumor cells and in cell lines expressing recombinant proteins (Figure 3) [3,41–43].

#### 2.3.1. WDSV rv-cyclin

**Figure 4.**
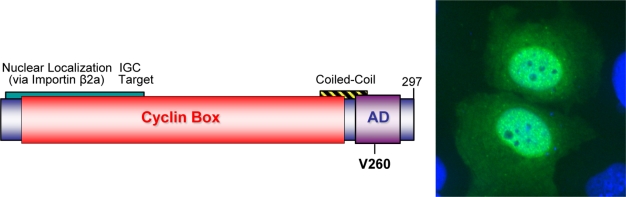
Model of rv-cyclin functional domains (Left) and localization in the nucleus in interchromatin granule clusters (Right). Cells were transfected with an rv-cyclin expression vector (pKH3-rv-cyclin) and stained with anti-rv-cyclin antisera and FITC-conjugated goat anti-rabbit IgG (green). Nuclei were stained with DAPI (blue). Magnification x400.

The WDSV *orf a* transcript is one of two multiply spliced transcripts detected during tumor development, suggesting a role in tumor formation [27,38]. Sequence comparisons of the WDSV and WEHV Orf A proteins identified the presence of a cyclin box fold so it was named “retroviral cyclin” or “rv-cyclin” [44]. The rv-cyclin sequences do not have sufficient homology to host cyclins (less than 20% linear sequence identity to any metazoan cyclin) to have resulted from a recent transduction of a cellular oncogene. The WDSV rv-cyclin is a 297 amino acid protein that, in addition to the cyclin box (a.a. 1–220), also contains an acidic transcription activation domain (a.a. 240–270) with a TAF9 binding motif (a.a. 257–266) in its extended C terminal region (Figure 4, left) [44–46]. In contrast, the shorter WEHV-1 and WEHV-2 rv-cyclins contain only the predicted cyclin box.

Whether detected as native protein in naturally infected tumor cells (Figure 3) or over-expressed in a variety of mammalian and piscine cell lines, the WDSV rv-cyclin is primarily localized in the nucleus and is concentrated in perichromatin fibrils and interchromatin granule clusters (IGCs; also known as nuclear speckles) (Figure 4, right) [3,43]. IGCs are defined by high concentrations of transcription and splicing factors, such as splicing component, SC35, and are at or near chromatin domains exhibiting high rates of transcription [47–52].

Rv-cyclin co-purifies and is co-precipitated with hyperphosphorylated forms of RNA polymerase II (RNA Pol II), p300, CBP, TATA-binding protein (TBP) and Mediator components [45,46,53]. The localization and physical association of rv-cyclin with components of transcription indicated a role in transcription regulation, a common function among complex retroviruses. Reporter assays of WDSV and SV40 promoters demonstrated that rv-cyclin may inhibit or enhance individual promoter activities in a cell-specific manner [45,53,54]. The WDSV rv-cyclin inhibits transcription from the WDSV promoter. Experiments using a transiently transfected reporter system determined that this inhibition works with a minimal core promoter and is independent of specific sequences in the enhancer region [53]. The transcription activation domain (AD) of rv-cyclin was first identified in studies addressing the function of rv-cyclin in transcription regulation using GAL4 fusions of rv-cyclin [45]. Fusion of the GAL4 DNA binding domain to rv-cyclin or to the isolated AD resulted in activation of transcription from a GAL4 responsive luciferase reporter. Mutation V260S within the AD eliminated activation of transcription from the GAL4 luciferase reporter. Inhibition of the WDSV promoter is also dependent upon the rv-cyclin AD and the V260S mutation relieves this inhibition [45]. Zhang and Martineau [54] also demonstrated rv-cyclin inhibition of promoter activity, however, they attributed this effect to the first 49 amino acids. The WDSV rv-cyclin AD co-precipitates with p300/CBP and the Mediator component Med23 (Sur2) and directly binds to TATA binding protein-associated factor 9 (TAF9) [46,53]. Binding of rv-cyclin to TAF9 is dependent upon amino acid V260 and binding was lost with a V260S mutation [46]. The conservative V260F mutation retains binding to TAF9 and function in reporter assays. The AD interferes physically and functionally with herpes simplex virus VP16-TAF9 contact and with the function of transcription factor, NF-κB, in transient transfection assays with an NF-κB-specific reporter [46,55]. This inhibition was found to be the result of the rv-cyclin AD’s ability to interfere with the function of the p65 activation domain, which is also dependent upon TAF9 binding. Together these studies indicate that the AD functions in both the activation and inhibition of host and virus gene expression. The negative regulation of the WDSV promoter and of NF-κB-dependent promoters, such as the type I interferon promoter in fish, is likely advantageous early in infection. These mechanisms may thwart the innate immune response and maintain low levels of virus expression during tumor development in order to avoid humoral and cell-mediated immune responses and the cytopathic effects associated with virus production.

The cyclin box fold is a protein-binding domain common to cyclins, TFIIB, and Retinoblastoma protein (pRb). Each contains two copies of the domain, which is characterized by a similar alpha-helical structure, but with remote linear sequence identity [56]. Alignments of the rv-cyclin with cyclins A, C, and D have been proposed based on combinations of sequence identity and proposed function [44,53,57]. The presence of the cyclin box fold suggests an interaction with a cyclin-dependent kinase (cdk), and a screen for such an interaction demonstrated an association of rv-cyclin with cdk8 [53]. Cdk8 and its partner, cyclin C are components, with Med12 and Med13, of the “cdk8 module” of the Mediator complex, which functions as a modifier of transcription. They are also highly conserved with 96% and 94% sequence identity between human and zebrafish cdk8 and cyclin C, respectively. Recent studies demonstrate a direct interaction of rv-cyclin with cdk8 by co-IP, GST pull down, and between purified, recombinant rv-cyclin and cdk8 [58]. The binding of the rv-cyclin to cdk8 does not appear to alter cdk8 substrate specificity, but does alter its nuclear localization, resulting in a tight association of a significant portion of the cdk8 pool with chromatin. Cdk8 is a positive regulator of transcription at chromatin via phosphorylation of serine 10 of histone 3, of transcription factor E2F1, and of serine 5 of the heptad repeat in the carboxy terminal domain of RNA Pol II, which increases the processivity of transcription elongation [59–61].

Cyclin C also partners with cdk3, an interaction that promotes phosphorylation of pRb to initiate G0/G1 transition and exit of quiescent cells from G0 [62]. Based on these observations an interaction of WDSV rv-cyclin with cdk3 was evaluated and it was found that rv-cyclin also interacts directly with cdk3 and activates cdk3 phosphorylation of pRb [58]. Using yeast (*Saccharomyces cerevisiae*) conditionally deficient in G1 cyclins (*Cln* genes) LaPierre *et al.* [44] demonstrated that WDSV rv-cyclin, but not WEHV rv-cyclins, restored cell growth. The interaction of WDSV rv-cyclin with cdk3 would explain the results of these studies. Cyclin C was first identified in complementation assays using yeast deficient in *Cln* genes [63–65]. Combined, these data suggest that WDSV rv-cyclin functions as an ortholog of cyclin C.

Identification of rv-cyclin as a structural and functional ortholog of a cellular cyclin, suggests a role in tumor development. In transgenic mice, expression of rv-cyclin from a keratin promoter was associated with severe squamous cell hyperplasia and dysplasia at sites of injury such as tail clipping [66]. In contrast, transgenic expression of WDSV rv-cyclin in zebrafish did not result in tissue proliferation even following tissue regeneration after fin clipping [67]. No increase in tumor incidence was seen in rv-cyclin transgenic fish exposed to N-ethyl N-nitrosourea (ENU), a chemical carcinogen, over that observed in control transgenic fish treated with ENU [67]. In a second study of rv-cyclin transgenic zebrafish in which rv-cyclin was expressed in the liver, no apparent tumors developed [68]. Treatment of fish with the chemical carcinogen 7,12-dimethylbenz [α] anthracene (DMBA) resulted in the development of liver tumors in 45% of rv-cyclin negative fish and 51% of the rv-cyclin transgenic fish [68]. Based on histopathology, the rv-cyclin negative fish had a higher incidence of malignant liver neoplasia than the rv-cyclin transgenic fish. Additionally, the time to development of tumor, after exposure to DMBA, was increased in rv-cyclin transgenic fish. Zhan *et al.* [65] suggest that rv-cyclin suppresses the development of liver tumors after chemical carcinogen exposure.

#### 2.3.2. WDSV Orf B

The WDSV Orf B protein, like rv-cyclin, is expressed during tumor development. WDSV Orf B localizes to the plasma membrane in structures consistent with focal adhesions and lamellapodia, and is found associated with actin stress fibers (Figure 3) [3,41]. WDSV Orf B directly interacts with the Receptor for Activated Kinase C (RACK1), a highly conserved adaptor protein that binds to activated, conventional isoforms of protein kinase C (PKC) [41]. The amino acid sequence of walleye RACK1 is 96% identical to human and mouse RACK1. RACK1 functions as an anchoring protein to stabilize PKC in an active conformation at the cell membrane [69,70]. PKC regulates signaling pathways that mediate cell proliferation and survival. In NIH-3T3 cells that stably express Orf B (NIH-3T3-Orf B), Orf B is found in a complex with PKCα [41]. Activated PKCα was only translocated to membranes after treatment of NIH-3T3 cells with PMA. In contrast, activated PKCα was present in membrane fractions from NIH-3T3-Orf B cells cultured without serum and without PMA stimulation. When cultured without serum, NIH-3T3-Orf B cells were able to proliferate. However, in the presence of the PKC inhibitor, bisindolymaleimide hydrochloride, the growth of these cells was significantly diminished [41]. Treatment of NIH-3T3-Orf B cells with concentrations of staurosporine known to induce apoptosis did not cause death of these cells, and in a screen to identify downstream targets of activated PKC, the pro-apoptotic protein, BAD, was found to be inactivated by phosphorylation at residues 112, 136 and 155, substrates of activated PKC and AKT signaling pathways [71]. Further studies showed that AKT is activated in NIH-3T3-Orf B cells, and that this activation also contributes to cell survival and functions to elicit a transformed phenotype [71]. Overall, these studies indicate that expression of Orf B leads to the activation of PKC and AKT signaling pathways and has the capacity to transform cells *in vitro*.

#### 2.3.3. WDSV Orf C

**Figure 5.**
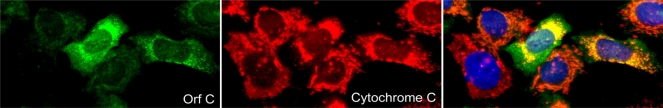
WDSV Orf C localizes to the mitochondria. Cells transfected with HA-tagged Orf C expression vector were stained with rabbit anti-HA antisera and a mouse monoclonal antibody that recognizes cytochrome c followed by staining with anti-rabbit IgG-FITC (green) or anti-mouse IgG-rhodamine (red). An overlay image of the staining is presented in the right panel. Nuclei were stained with DAPI (blue).

The third novel accessory protein of WDSV, Orf C, is encoded by an open reading frame that lies 5’ proximal to *gag* (Figure 2) [5]. After the end of the *orf c* reading frame there are seven base pairs prior to the initiation of the *gag/pro/pol* polyprotein. No specific *Orf C* transcript has been identified, so the protein is presumed to be translated from the full-length WDSV genomic mRNA, which is present at high levels during tumor regression [38]. The Orf C protein is present in regressing tumors and can be detected by western blot and in regressing tumor sections and in tumor explant cells by immunofluorescence (Figure 3) [3,42]. The specific cytoplasmic localization of Orf C in mitochondria was identified in cells transiently transfected with an Orf C expression plasmid (Figure 5) [42]. The co-localization of Orf C with cytochrome c in mitochondria was associated with their perinuclear clustering and loss of mitochondrial membrane potential, which was confirmed by the inability of Orf C-positive mitochondria to retain the vital dye, Mitotracker. The mechanism of localization of the Orf C protein was predicted to be dependent upon an amphipathic alpha helix at position 78 to 89 [72], but efforts to knockout this mitochondrial targeting motif have been unsuccessful [73].

**Figure 6.**
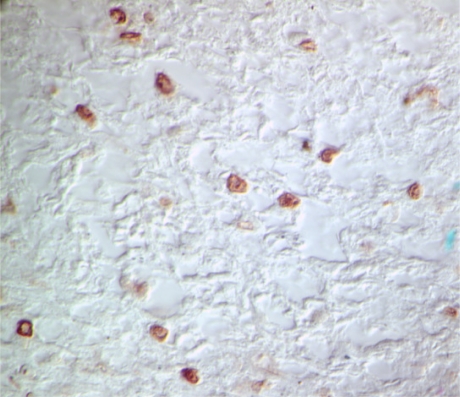
TUNEL staining of regressing dermal sarcoma. HRP mediated 3,3′-Diaminobenzidine (DAB) deposition (brown) in nuclei after biotin-dUTP labeling of chromosomal DNA strand breaks.

The outcome of the over-expression of Orf C in piscine and mammalian cells is the induction of apoptosis [42]. The role of apoptosis in tumor regression was demonstrated by terminal deoxynucleotidyl transferase biotin-dUTP nick end labeling (TUNEL) of sections of regressing tumors (Figure 6). This assay detected a significant number of apoptotic nuclei (brown precipitate) in a typical regressing tumor. These data suggest a direct role for the Orf C protein as an oncolytic protein and indicate that tumor regression, like the tumorigenic process, may be largely controlled by WDSV accessory proteins. Furthermore, both processes—the formation and growth of the tumor mass and its regression and shedding from the host—are integral to successful WDSV replication and dissemination.

## 3. Conclusions

WDSV is exceptional among tumor virus models in that it has incorporated tumorigenesis and tumor regression into its life cycle and coordinates these processes with the seasonal spawning of walleye. After infection with WDSV there is a period of restricted virus expression, associated with tumor progression followed by a switch to overt virus production and tumor regression. The fully developed sarcoma with its high levels of cell-associated virus serves as a vessel for virus transmission in that the tumor mass is shed into the surrounding water during a period of maximum fish-to-fish contact. This final step serves in the efficient transmission of virus to naïve animals in an aquatic environment. Epidemiology studies suggest that the process occurs once in the lifetime of the host indicating that fish are resistant to development of tumors in subsequent years.

The functions of the WDSV rv-cyclin and Orf B proteins in this process appear to be twofold: constrain virus replication and drive tumor formation. Clear evidence exists for down regulation of the WDSV promoter by rv-cyclin. This protein also has the capacity to play a direct role in transformation by regulation of host gene transcription and promotion of the entry of quiescent cells into cycle via cdk3 activation. The rv-cyclin may also interfere with innate immune mechanisms by blocking NF-κB and interferon transcription. Orf B protects cells from apoptotic stimuli, a function that is crucial in tumorigenesis, and its insinuation into signal transduction pathways also affords it mechanisms for a direct role in cell proliferation. Expression of Orf B alone is sufficient for transformation of cells *in vitro*. rv-cyclin and Orf B probably work together to cause the rapid and efficient formation of dermal sarcomas (greater than 85% of infected animals in three months post-infection). The Orf C protein is expressed in conjunction with the production of infectious virus and the initiation of tumor regression. Orf C induces apoptosis *in vitro*, and Orf C is present in regressing tumor cells when apoptosis is apparent and virus expression is greatly increased. Other mechanisms, particularly high levels of WDSV UVD, probably contribute to virus production and to tumor cell death. The host immune response likely contributes to rejection of the tumor mass and subsequent protection against new tumors.

The virus and host have established a fine balance in order to orchestrate this seasonal process, thus there is little selective pressure on the virus, as is apparent from the stability of its genome over time. The significance of this aspect of the model is that viral mechanisms that manipulate the host cell are highly refined and their targets are evolutionarily conserved. WDSV must be responsive to certain host restriction factors that block infection or replication and yet it must control restriction factors in the target tissue during infection, tumor growth, and virus replication. Whether it be restriction factors, signal transduction pathways, transcription and splicing regulation, or apoptosis induction, WDSV has evolved a great deal of control from a very small coding capacity and it leaves behind a fit and reproductive host.
